# New Characterization of Lipedema Stages: Focus on Pain, Water, Fat and Skeletal Muscle

**DOI:** 10.3390/life15091397

**Published:** 2025-09-03

**Authors:** Sara Al-Ghadban, Jane V. Evancio, Paula E. F. Alfiscar, Karen L. Herbst

**Affiliations:** 1 Department of Plastic Surgery, Maxillofacial & Oral Health, University of Virginia, Charlottesville, VA 22903, USA; 2 The Roxbury Institute, Tucson, AZ 85715, USA

**Keywords:** lipedema, stages, pain, swell, heaviness, fatigue

## Abstract

Lipedema is a chronic, progressive adipose connective tissue disorder characterized by symmetrical, disproportionate fat accumulation, typically affecting the lower extremities and arms, accompanied by pain, swelling, and a sensation of heaviness. This study introduces intermediate Stages 1.5 and 2.5 to the established lipedema classification (Stages 1, 2 and 3), and other affected areas, based on physical examination, a questionnaire, and photographic documentation. Bioelectrical Impedance Spectroscopy (BIS) was employed to quantify total body water (TBW) across stages. A significant and linear increase in BMI was observed from Stage 1 to 3, correlating with increased reported pain and heaviness in the thighs, calves, and upper arms. Systemic symptoms of brain fog, debilitating fatigue, and hypothermia were significantly prevalent. TBW demonstrated a significant, stage-dependent increase in the lower extremities. Adipose tissue accumulation over the knees and feet significantly increased with lipedema stage. In contrast, shin involvement was evident in early stages and remained consistently elevated throughout later stages. Skeletal Muscle Mass (SMM) exhibited a significant increase across lipedema stages, positively correlating with fat mass (FM) in Stage 3. This study elucidates previously underrecognized clinical features and distribution patterns of lipedema, offering a refined staging system to improve understanding of its progression and burden.

## 1. Introduction

Lipedema is a disorder of connective (adipose) tissue that affects women worldwide. Women with lipedema suffer from pain, unexplained weight gain, a feeling of heaviness in the legs, muscle weakness, sleep apnea, and reduced quality of life [1,2,3,4,5,6,7,8]. Clinicians often misdiagnose lipedema as obesity, lymphedema or chronic venous disease [1,9,10,11]. Although these disorders share significant overlap in their symptomatology, a comprehensive physical exam is crucial to identify women suffering from lipedema.

Lipedema is a progressive connective tissue disorder affecting fat, lymphatics, and microvasculature. The current stage classification is established and has been reviewed in the Standard of Care for Lipedema in the US published by Herbst et al. in 2021 [1]. However, in photographic examples of lipedema, we observe abrupt increases in tissue amount, texture and shape between Stage 1 and 2, and Stage 2 and 3, suggesting that the current three-stage classification may not fully capture changes in lipedema tissue; disease progression occurs along a continuum, not as discrete jumps from 1 → 2 → 3. Based on systematic physical examination findings and photographic documentation from a single specialized clinic, we propose the addition of two intermediate stages—Stage 1.5 and Stage 2.5. Our rationale stems from repeated clinical observations that some patients do not fit neatly into the existing stage definitions but instead display overlapping features, reflecting transitional phenotypes from smooth → textured → lobular.

These transitional forms allow more precise morphological description in patients with mixed features and may represent biologically meaningful phases that are important for staging disease severity, guiding treatment decisions, and improving participant stratification in research studies. By introducing Stage 1.5 and Stage 2.5, we aim to reduce staging disagreements in patients with overlapping features, improve interobserver reliability, improve clinical reproducibility and study stratification, enhance lipedema classification enabling clinicians to better monitor disease progression, identify at-risk patients earlier, and support more precise grouping in clinical research.

Similar half-stage models have been adopted in lymphedema (ISL 2a/2b) to improve phenotyping and management [12].

In addition to redefining the clinical staging, it is important to address symptoms and signs that significantly impact the quality of life in women with lipedema but are often overlooked or underreported [13,14]. These symptoms include brain fog, extreme fatigue, joint hypermobility, and qualitative reporting of hypothermia of the skin. Joint hypermobility, a feature commonly associated with Ehlers-Danlos Syndrome (EDS), is frequently reported by women with lipedema [15,16], yet its association to lipedema is not understood. Thus, we aim to explore joint hypermobility across different stages of lipedema using the Beighton score to better understand its prevalence and potential clinical significance.

Previous studies have shown that lipedema tissue contains a higher amount of fluid outside the cells compared to healthy tissue [16,17,18,19]. In this study, we quantify intracellular fluid (ICF), extracellular fluid (ECF), and total body water (TBW) by bioimpedance spectroscopy (SOZO), and examine their relationship with SMM and FM in women with lipedema. By examining these fluid compartments alongside SMM and FM, we aim to better understand the complex interplay between fluid accumulation, muscle, and adipose tissue in lipedema. This approach may provide new insights into the pathophysiology of lipedema and offer objective biomarkers to improve diagnosis and staging.

Overall, this article highlights underrecognized features of lipedema, focusing on updated staging, newly identified affected areas, and highly prevalent signs and symptoms, to improve understanding of the disease burden. By comparing intermediate stages with the established stages, we provide a more comprehensive view of disease progression, leading to improved diagnostics and management strategies tailored to the specific needs of patients at each stage.

## 2. Materials and Method

### 2.1. Chart Review

Participants included in this study were sequential patients diagnosed with lipedema (n = 102) in an outpatient clinic seen by a single provider (KLH) in 2024 over a period of 6 months (Table 1). This study is considered exempt from institutional review board oversight because data analyzed were collected during usual medical visits.

Physical exams utilized an itemized head to foot palpation and visual exam by a single provider (KLH) modified from the criteria of Wold et al. [20]. Hypermobile joints were assessed by the Beighton score [21] with a score of 5 out of 9 possible points considered positive.

Medical history was obtained verbally but also through a questionnaire completed by patients on the Klara Technologies, LLC platform (Boca Raton, FL, USA). As part of this questionnaire, extreme fatigue was defined as *lingering tiredness that is constant and limiting; in other words, unexplained, persistent, and relapsing exhaustion* [22]. Brain fog was described as *a constellation of symptoms including reduced cognition, inability to concentrate and multitask, as well as loss of short and long term memory* [23].

### 2.2. Bioimpedance Spectroscopy (BIS)

Fluid/water of the whole body, the legs and the intracellular and extracellular fluid in the legs, and kg FM, kg fat free mass (FFM) and kg SMM were assessed by BIS (SOZO applications, Impedimed, Brisbane, Australia) [24] as published previously [18]. Bilateral L-Dex analysis was used to measure the risk of lymphedema in the legs, representing the ratio of measured impedance between the arms and legs, with the legs being the at-risk limbs and the arms the lower risk limbs [18]. Bioelectrical Impedance Analysis (BIA) at a 50 kHz frequency yields two primary raw measures: resistance (R) and reactance (Xc). The BIS Phase Angle (PhA) is mathematically defined as the arctangent of the ratio of reactance to resistance (PhA = arctan (Xc/R). PhA serves as a quantitative indicator of cell membrane integrity and a robust surrogate marker for detecting inflammatory and oxidative stress states [25]. During periods of inflammation and oxidative stress, elevated levels of reactive oxygen species (ROS) induce damage to cell membranes, concurrently altering fluid distribution between the intracellular and extracellular compartments. This disruption directly impacts the capacitive properties of cell membranes. Consequently, diminished cellular structural integrity and increased cellular apoptosis or necrosis are correlated with lower PhA values, while enhanced cellular function and overall cellular health are associated with higher PhA values.

### 2.3. Hypothermia

Hypothermia of the skin was assessed by qualitative thermography with a FLIR camera (FLIR ONE PRO LT iOS Thermal Camera, Teledyne FLIR, Teledyne Technologies, Thousand Oaks, CA, USA), where the color magenta represents an area of lower temperature and was denoted as hypothermia, and yellow to white an area of warmer temperature, determined as an area without hypothermia.

### 2.4. Skeletal Muscle Mass Index (SMMI)

Skeletal muscle mass index (SMMI) was calculated by dividing the appendicular muscle mass of the legs by the height (m^2^).

### 2.5. Statistics

Data are presented as mean ± standard error of the mean (SEM). Differences between measures across stages were evaluated using paired parametric tests (3 to 4 evaluations per subject). A simple linear regression was performed using the equation Y = mX + b. Statistical significance was set at *p* < 0.05. All analyses were conducted using GraphPad Prism version 10.3.1 (GraphPad Software, Boston, MA, USA; www.graphpad.com, accessed on 25 November 2024).

A post hoc power analysis was performed using one-way ANOVA to assess differences in Age and BMI across the five lipedema stages. Effect sizes were large, with Cohen’s f = 2.23 for Age and f = 4.40 for BMI. Given the total sample size of 102 participants and α = 0.05, the achieved statistical power for both variables was 1.00, indicating that the study was more than adequately powered to detect group differences.

## 3. Results

### 3.1. Characteristic of Lipedema Stages

**Figure 1:**
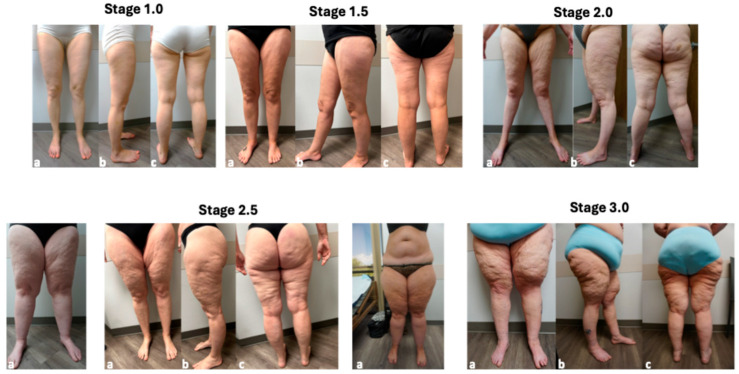
Stages and features of lipedema. Front (**a**), side (**b**) and back (**c**) pictures of women with lipedema Stage 1.0. 1.5, 2.0, 2.5 and 3.0. Three patients are presented for stage 2.5 to show the variety of presentations. All photos are of women with lipedema of the Allen and Hines type [27] with smaller ankles versus the Moncorps Type with a larger ankle cuff [28].

The classification of stages including half stages are described below (Table 2) and presented as photos (Figure 1). As the number assigned to a stage increases, changes in skin texture become more extensive reflecting fibrosis of skin and tissue fibers [1] and fibrosis and greater amounts of glycosaminoglycans in the interstitial space [1,26] along with increased amounts of subcutaneous tissue and size of masses in the tissue.

### 3.2. BMI Significantly Increases in Stages 2 and 3

**Figure 2:**
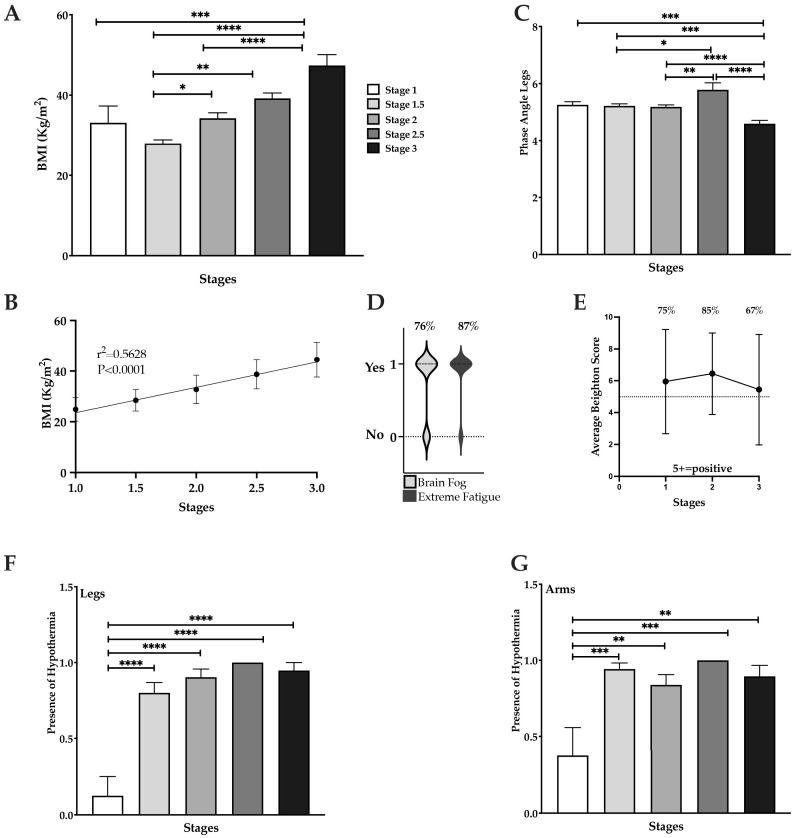
**Multiple measures in lipedema patients.** (**A**). BMI significantly increases by stage. (**B**). Correlation between BMI and stages. (**C**). Phase Angle, a marker of inflammation, was significantly lower in Stage 3. (**D**). Percentage of women with lipedema with brain fog and extreme fatigue. (**E**). Beighton scores and percent positive (Beighton score ≥ 5). (**F**). Percent affected by hypothermia in legs with Stage 1 significantly lower than other stages; (**G**). Percent affected by hypothermia in arms with Stage 1 significantly lower than other stages; * *p* ≤ 0.05, ** *p* ≤ 0.01, *** *p* ≤ 0.001, **** *p* ≤ 0.0001.

Our data confirm that BMI increases gradually and significantly from Stage 1 to Stage 3 (*p* < 0.0001, Figure 2A) with a significant increase in BMI between stage 1.5 and Stage 2 (*p* = 0.001), Stage 1.5 and Stage 2.5 (*p* = 0.0002), and Stage 1.5 and Stage 3 (*p* < 0.0001). The increase in BMI across stages was positively and significantly correlated (r^2^ = 0.5628; Figure 2B), suggesting that body mass is tightly linked to disease severity. In addition to the increase in BMI, women with lipedema had moderate (Stage 1–2) to high (Stage 3) inflammation in the legs, assessed by PhA (Figure 2C).

### 3.3. High Prevalence of Fatigue, Brain Fog, Hypermobility, and Hypothermia in Lipedema

Currently, lipedema is not widely considered a systemic disease in clinical practice and research [29,30,31]. However, in our study, women with lipedema had systemic findings including self-reported extreme fatigue (87%) and brain fog (76%) (Figure 2D), and were found on exam to have increased joint mobility in 75% or more patients, as assessed by the Beighton score for Stage 1 (75% affected) and Stage 2 (85% affected); this value dropped to 67% for women with Stage 3 lipedema (Figure 2E), but these women were also older than the other stage groups (Table 1).

Hypothermia by qualitative thermography in both legs (Figure 2F, *p* < 0.0001) and arms (Figure 2G, *p* < 0.001) was significantly higher in Stages 1.5, 2, and 3 compared to Stage 1.

### 3.4. Pain, Swelling and Heaviness in Lipedema

**Figure 3:**
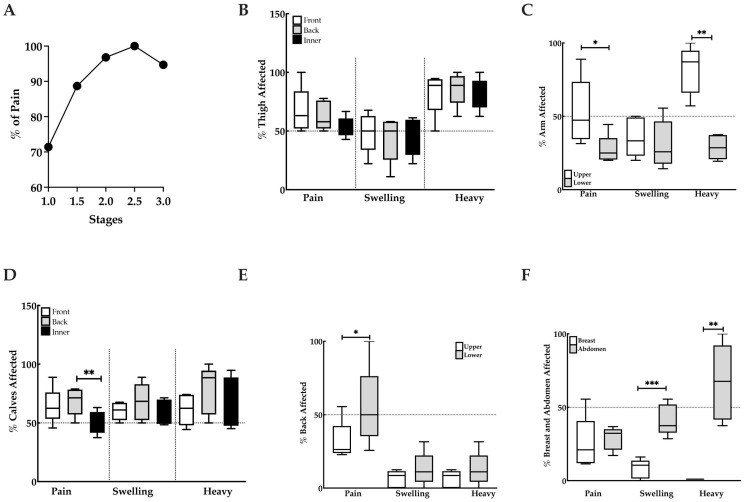
Pain, swelling and heaviness in lipedema tissues. (**A**). Pain increases with stage. B. Representation of total pain, swelling and heaviness of the thighs (**B**), arms (**C**), calves (**D**), back (**E**) and breast and abdomen (**F**). * *p* ≤ 0.05, ** *p* ≤ 0.01, *** *p* ≤ 0.001.

Quantitative measures of lipedema tissue increase with stage simultaneously with qualitative measures of pain, swelling and tissue heaviness. Our data demonstrate that 70% of women with Stage 1 lipedema experience pain, while 30% do not, suggesting that in early stages, pain is not necessary for a diagnosis of lipedema. The percent of women that experience pain with lipedema increased gradually with Stage 2 and 3. Interestingly, 90% of women with Stage 1.5 lipedema experience pain and 100% with Stage 2.5 lipedema. It would be expected that all women with Stage 3 lipedema have pain, but this number was only 95%. (Figure 3A).

The thighs, upper arms, calves, and lower back are the most painful areas in women with lipedema (Figure 3B–E). Pain in these affected areas correlates with heaviness in the tissues; however, swelling was only observed in the upper arms and calves (Figure 3C,D), suggesting another cause for pain in the thighs and low back other than swelling. Interestingly, women with lipedema experienced significant swelling and heaviness in the abdomen as compared to the breast (Figure 3F). Women were less likely to complain of pain in the lower arms, abdomen or breast areas (Figure 3C,F).

### 3.5. Leg Fat Increases with Advancing Stages of Lipedema

**Figure 4:**
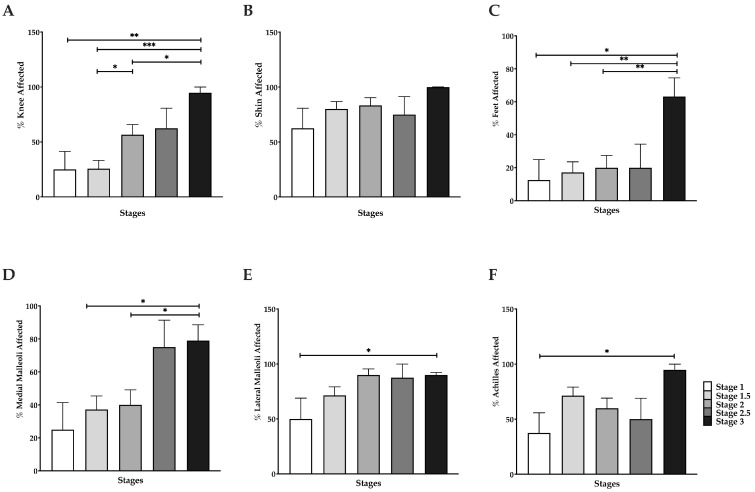
Percentage of women with fat in multiple locations on the leg by lipedema stage. (**A**). Covering patella; (**B**). Covering shin. (**C**). Anterior foot; (**D**). Medial malleolus; (**E**). Lateral malleolus; and (**F**). Around Achilles tendon. * *p* ≤ 0.05, ** *p* ≤ 0.01, *** *p* ≤ 0.001.

In addition to the increase in thigh fat observed in lipedema patients, we detected increases in fat covering the patella (knee), anterior shin and anterior foot with increasing stage (Figure 4A,C). Compared to Stage 1 or Stage 1.5, fat that grew over the patella increased significantly in patients with Stage 3 lipedema (*p* = 0.0317 and *p* < 0.001, respectively). Additionally, there was a 40% increase in the number of lipedema patients that had fat covering the patella from Stage 2 to Stage 3 (*p* = 0.0314), as well as from Stage 1.5 to Stage 2 (*p* = 0.0441, Figure 4A). Fat accumulation on the anterior foot affected 50% more patients from Stage 1 to Stage 3 (*p* = 0.0357), and for Stages 1.5 and 2 to Stage 3 (*p* = 0.0016 and *p* = 0.0014, respectively; Figure 4C). Fat that covers the shin appears to be a relevant finding in all stages where the percent of women whose shins were completely covered by fat tissue was high in early stages and remained high in later stages so that there was no significant difference between the stages for this marker of lipedema (Figure 4B).

Additionally, our data show an increase in the percentage of fat around the medial malleolus from Stage 1.5 to Stage 3 (*p* = 0.02, Figure 4D), and from Stage 2 to Stage 3 (*p* = 0.048, Figure 4D). We also observed a significant increase in fat around the lateral malleolus between Stage 1 and Stage 3 (*p* = 0.0138, Figure 4D). Finally, fat accumulation around the Achilles tendon significantly increased from Stage 1 to Stage 3 (*p* = 0.026, Figure 4F).

### 3.6. Total Body Water (TBW) Increases with Stage in Lipedema Tissue

**Figure 5:**
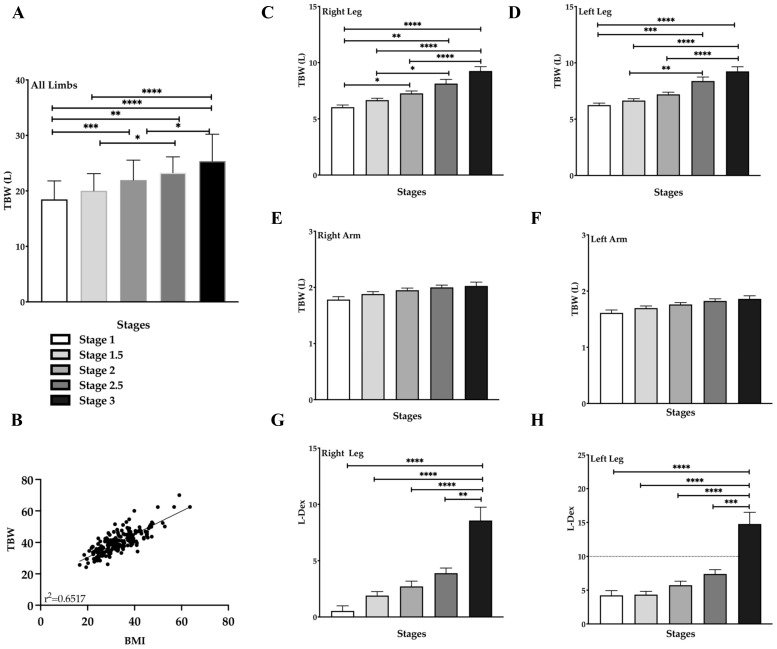
Total Body Water (TBW) Increases with Stage in Lipedema (bioimpedance spectroscopy). (**A**). TBW in four limbs across Lipedema stages. (**B**). Correlation between TBW and BMI. (**C**,**D**). TBW in right and left legs, respectively. (**E**,**F**). TBW in right and left arms, respectively. (**G**,**H**). Lymphedema risk by L-Dex increases by stage in the right and left leg, respectively. The dotted line indicates the upper limit of the normal range. * *p* ≤ 0.05, ** *p* ≤ 0.01, *** *p* ≤ 0.001, **** *p* ≤ 0.0001.

Studies have demonstrated that lipedema tissue contains higher water and sodium levels compared to non-lipedema tissue [16,17]. Our study of women with lipedema found that total body water (TBW) in the four limbs significantly increased across stages: Stage 1 to Stage 2: *p* = 0.0036; Stage 2 to Stage 3: *p* = 0.028; Stage 1 to Stage 3: *p* < 0.0001 (Figure 5A). In addition, TBW increased between intermediate stages as well, particularly between Stage 1 and Stage 2.5 (*p* = 0.0021), and between Stages 1.5 and 2.5 compared to Stage 3 (*p* = 0.0341 and *p* < 0.001, respectively; Figure 5A). Interestingly, the increase in TBW positively correlated with an increase in BMI (*p* < 0.001, Figure 5B). TBW in the legs significantly increased between stages for both the right (Figure 5C) and left (Figure 5D) legs. However, no significant differences in TBW were observed in the arms across all stages (Figure 5E,F). Finally, there was a significant increase in the potential risk of lymphedema (L-Dex) in Stage 3, compared to all other stages, for both the right (Figure 5G) and left legs (Figure 5H).

### 3.7. Decrease in ICF/ECF in the Left Arm & Legs in Lipedema Stage

**Figure 6:**
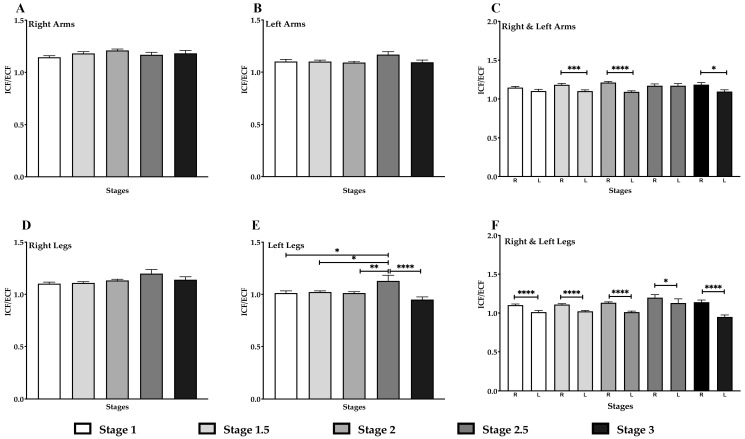
Ratio of ICF/ECF in Lipedema. (**A**,**B**). No difference between arms in lipedema stages. (**C**). Comparison between the right and left arms showing a decrease in the left arms compared to the right arms in Stage 1.5, 2 and 3. (**D**,**E**). Significant increase in left legs in stage 2.5 compared to other stages. (**F**). Comparison between the right and left legs showing a decrease in left legs compared to right legs in all stages. * *p* ≤ 0.05, ** *p* ≤ 0.01, *** *p* ≤ 0.001, **** *p* ≤ 0.0001.

Higher values for ECF (lower ICF/ECF) suggestive of increased fluid in the interstitial space (or in blood vessels), could indicate edema in lipedema tissue. In this study, no significant difference in the ICF/ECF ratio was observed in the right or left arms across all stages (Figure 6A,B). However, a significant decrease in the ICF/ECF ratio was found when comparing the right and left arms in stages 1.5 and 2 versus 3 (*p* < 0.001 and *p* < 0.05, respectively; Figure 6C). No significant differences in the ICF/ECF ratio were detected in the right leg across all stages (Figure 6D). While there appeared to be a higher ICF/ECF in the left leg in Stage 2.5, this value may reflect the small number of individuals in this group (n = 8). When comparing the ICF/ECF ratio between the right and left legs, a significant decrease was observed in the left leg across all stages (*p* < 0.0001; Figure 6F), suggestive of higher ECF.

### 3.8. Skeletal Muscle Mass (SMM) Increases with Fat Mass in Lipedema Stage 3

**Figure 7:**
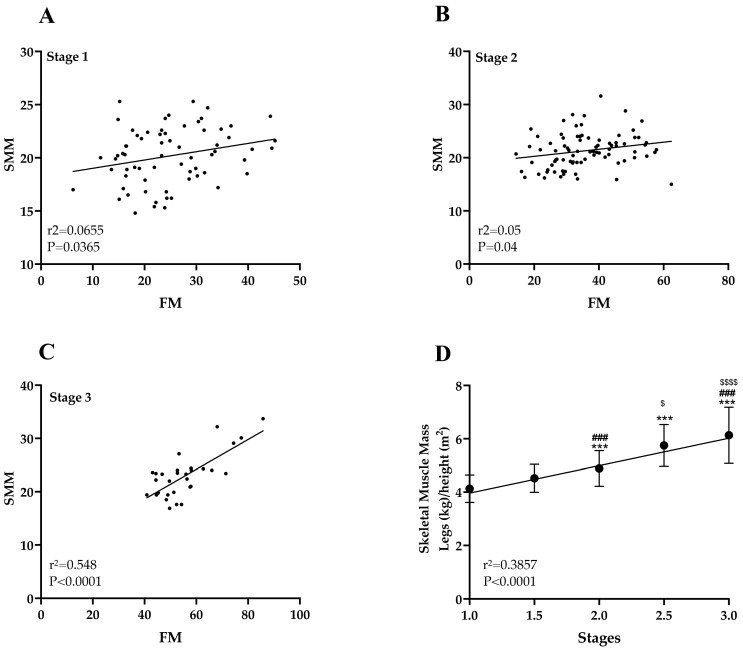
Correlation between skeletal muscle mass (SMM) and fat mass (FM) in the legs of lipedema stages 1–3. (**A**,**B**). There was no significant correlation between SMM & FM in Stage 1 (**A**) and Stage 2 (**B**). (**C**). Significant correlation between SMM and FM Stage 3. (**D**). Significant increase in SMM in the legs to height in all lipedema stages, including half stages. *** Significant difference from Stage 1; ^###^ Significant difference from Stage 1.5; ^$^ and ^$$$$^ Significant difference from Stage 2. *** *p* ≤ 0.001; ^###^ *p* ≤ 0.001, ^$^ *p* ≤ 0.05, ^$$$$^ *p* ≤ 0.0001.

Muscle weakness can contribute to mobility issues in lipedema patients, potentially impacting quality of life and physical function [17]. In this study, we observed that skeletal muscle mass (SMM) in all limbs significantly increased in parallel with fat mass (FM) in women with stage 3 lipedema (r^2^ = 0.548, *p* < 0.0001; Figure 7C). This finding suggests a possible compensatory mechanism, where muscle mass increases to support the excess adipose tissue weight. Additionally, the ratio of leg SMM to height (SMI) increased progressively across all stages of lipedema (r^2^ = 0.3857, *p* < 0.0001; Figure 7D), indicating muscle mass increases alongside disease progression.

## 4. Discussion

Women with lipedema present with the classic asymmetric changes in connective adipose tissue often during puberty. Over time, due to life changes of pregnancy, menopause, weight gain and/or additional inflammatory conditions, tissue changes lead to the development of a distinct clinical stage. The classic three stages, however, have dramatic differences between them in the amount and shape of the tissue [1], suggesting that extensive tissue changes occur between stages, and that additional stages are needed. In this paper, we propose two intermediate lipedema stages—referred to as Stage 1.5 (intermediate between Stages 1 and 2) and Stage 2.5 (intermediate between Stages 2 and 3).

These new stages provide an opportunity for a better understanding of how increased weight affects lipedema tissue, and we hope, focuses research effort on understanding changes in skin fibrosis (especially Stages 1–1.5), and the location of lobules as they first form (Stage 2.5). We have seen fibrotic changes in the skin in Stage 1.5 that affect only the superior portion of the thigh, or only the inferior portion of the thigh, but also asymmetry with the lateral thighs affected ¾ of the way down, but the anterior thigh only partially affected. The questions that these findings point out are, why is there fibrosis in the skin and underlying tissue, and how does the location of the fibrotic changes help us better understand lipedema. For example, we think that there is often a fasciitis of the iliotibial (IT) band on the lateral thigh that results in fibrosis of the overlying skin due to its proximity in this area, in agreement with others [4]. This is confirmed when nodules are palpated in this area, and the area is tender to palpation in all stages.

Identification of lobules as they form (Stage 2.5) is important for the prevention of fluid collection in these dependent areas that can lead to inflammation, fibrosis and further growth of the lobules that can eventually impair mobility and increase the risk of lymphatic damage and cellulitis.

Our data supports the addition of new intermediate stages in that both BMI and skeletal muscle mass demonstrated a significant and linear increase across all lipedema stages, with the newly introduced intermediate stages (1.5 and 2.5) fitting precisely within this progressive continuum.

Lipedema tissue is known for symptoms such as pain, swelling, and tissue heaviness [1,6,10,32,33,34,35,36,37,38,39,40]. The identification and association of these symptoms by individual locations are rarely found in published literature, especially by stage. Our data demonstrated that the most affected area of the body was the calves with over 50% of the women having pain, swelling and a feeling of heaviness in this area of the body; no other area of the body was as affected. The lower aspect of the great saphenous vein and the small saphenous vein are in the calves. A Swiss study found that 86.2% of women with lipedema had chronic venous disease [38]. While we did not correlate vein disease with signs and symptoms in our study, these data suggest that the calves may be more affected because of the increased pressure in the veins upon standing, compounded by chronic vein disease, leading to increased fluid in the tissue. We agree that vein disease is an important co-morbidity in women with lipedema.

Some publications require pain for a diagnosis of lipedema [41]. Our data show that 30% of women with Stage 1 lipedema do not have pain, whereas 100% of women with Stage 2.5 lipedema have pain. In early stages, especially in young women, we should be aware that classic lipedema pain may not be present. Interestingly, not all women with Stage 3 lipedema had pain. Previous data suggests that numbness is present in women with Stage 3 lipedema [42] therefore the lack of pain may represent underlying neuropathy [6].

We assessed areas not classically associated with lipedema such as the abdomen, back, and breasts and found that the low back in women had more pain than the upper back and that women with lipedema experienced heaviness of the abdomen. While these symptoms could be attributed to spinal issues or obesity respectively, we should consider whether lipedema tissue is limited to appendicular areas or if it is a systemic disease. Although lipedema primarily affects subcutaneous adipose tissue, emerging evidence shows systemic inflammatory and metabolic markers that challenge its classification as solely localized. However, it is not yet widely recognized as a systemic disease in clinical guidelines. Studies report elevated circulating cytokines (IL-11, IL-28A, IL-29) [29,33], increased M2 macrophages influencing tissue and vascular changes [35], and metabolomic alterations including VEGF and sphingolipids [31], supporting possible systemic involvement.

Further supporting this concept, we found that most women with lipedema had extreme fatigue and brain fog, the latter being a persistent cognitive impairment syndrome. Recent research by Kunzová et al. supports this association, identifying brain fog as a symptom reported by participants with lipedema [13]. Brain fog is found in association with long COVID due to brain inflammation, cognitive decline, disrupted sleep patterns, and nutritional and mental health deficiencies, where impaired glymphatic function was associated with neurological dysfunction [43]. The fact that RNA and protein from the tissue of women with lipedema highlighted mitochondrial function and oxidative phosphorylation [31], and that mitochondrial complex dysfunction is associated with brain fog [44] suggests that we should be investigating glymphatic and mitochondrial function in women with lipedema.

Phase angle significantly decreased across stages except for Stage 2.5. These data suggest that inflammation increases with stage. However, recent data found a decrease in inflammation in the tissue and no obvious inflammatory marker in the blood of women with lipedema [31]. It may be that other pathways associated with tissue damage are altered in women with lipedema. The decreased phase angle with stage encourages us to continue to look for whatever is causing fibrosis in the lipedema tissue including oxidative stress detected by lower phase angles [25] and found to be altered in multiple studies [31,45].

Abnormal thigh fat among women with lipedema is well documented [10,46]; however, our study reveals that fat accumulation also develops over the knees, around the ankles and on the feet in later stages, while fat grows over the shin in women with Stage 1 lipedema as well as all other stages suggesting that fat over the shin is an early and definite sign of lipedema tissue. Fat was found around the medial malleolus, lateral malleolus and the Achilles tendon more often in women with Stage 3 lipedema but significantly less so in Stage 1, therefore ankle fat or the ankle cuff is more of a late finding in lipedema. These results indicate that fat deposition in lipedema extends beyond the classic thigh regions, highlighting the importance of comprehensive anatomical assessment of the leg in clinical evaluation and staging.

Women with lipedema experienced peripheral hypothermia in both the legs and arms, which becomes more noticeable in the later stages of the condition where the amount of adipose tissue is increased. Women with Stage 1 lipedema were less affected with hypothermia due to having less adipose tissue on their bodies. While this phenomenon has been mentioned in the clinical characteristics of lipedema in previous studies [1,5], it has never been quantified across different stages.

Our study corroborates previous findings showing that lipedema tissue contains higher water (and sodium) levels compared to healthy, non-lipedema tissue [3,17,18,47]. TBW in the four limbs significantly increased across lipedema stages—particularly between stage 1 and stage 2.5, and between stages 1.5 and 2.5. TBW in the legs also significantly increased across stages, for both the right and left legs. This increase in TBW in the legs was supported by elevated L-Dex scores, indicating a higher potential risk of lymphedema in Stage 3 compared to all other stages, for both the right and left legs. Interestingly, TBW was positively correlated with BMI and the ICF/ECF ratio, suggesting that increased adipose tissue led to increased fluid retention that may contribute to the edema observed in lipedema (Figure S1). No significant changes in the ICF/ECF ratio were observed in the right leg across all lipedema stages. In contrast, the left leg showed a decrease suggestive of more ECF in women with Stage 3 lipedema. When comparing the right and left legs, a significant reduction in the ICF/ECF ratio was observed in the left leg across all stages when compared to the right leg. These data are consistent with data found by Dean et al. where the left leg was affected by edema more than the right in all forms of lymphedema including lipedema with lymphedema due to the pelvic anatomy on the left side consistent with a subclinical May-Thurner syndrome [48].

No significant difference in TBW was observed between stages in the arms. Similarly, no significant changes in the ICF/ECF ratio were observed in the right or left arms across all lipedema stages. It may be that the legs accumulate more fluid than the arms primarily because they are constantly fighting against gravity over longer venous and lymphatic pathways, and are more prone to venous insufficiency, which creates a continuous predisposition for fluid filtration and retention.

When comparing the ICF/ECF ratio between arms, a significant decrease in the left arm was observed at stages 1.5, 2, and 3. There was therefore likely more ECF compared to ICF in the left arm compared to the right arms of women with lipedema. Others found that right limbs drain fluid more effectively than left, likely due to a combination of handedness and anatomical variation [49], which would explain the lower ICF/ECF ration in the left arm compared to the right arms of women with lipedema.

Muscle weakness can contribute to mobility issues in lipedema patients, potentially impacting their quality of life and physical function [1,2,24]. In this study, we observed that skeletal muscle mass in all limbs significantly increased in parallel with fat mass in women with stage 3 lipedema. This finding suggests a possible compensatory mechanism, where muscle mass increases to support the excess adipose tissue. However, we have to question the functionality of that muscle because the time it took women with primarily Stage 2 lipedema to complete a timed up and go (TUG) test was similar to the mean for 80–84-year-old women in a meta-analysis of TUG data [49]. Lipedema blood and tissue show markers of inflammation [4,34,35,36,50,51]. In later stages, lipedema fat can grow around muscle suggesting that inflammation may also affect muscle tissue [17]. Muscle damage results in vasodilatation and increased permeability of blood vessels secondary to inflammatory mediators resulting in swelling. In BIS, an increase in tissue water content is linked to a decrease in resistance [52]. Thus, the increase in body water, seen in our study, could cause an overestimation of muscle mass when using a whole-body model of BIS. Furthermore, the apparent increase in muscle mass in lipedema may reflect non-functional components such as intermuscular fat, edema, and fibrosis rather than true hypertrophy. These changes can lead to overestimation of muscle mass on tools like BIS. Reduced mobility due to pain, joint hypermobility, and fat accumulation may lead to muscle deconditioning, explaining the paradox of increased mass with decreased strength and function. These observations highlight the complex interplay between fat and muscle tissue in lipedema and underscore the need for holistic approaches to manage both fat accumulation and muscle function in the disease’s progression.

Finally, this study adds to data in the literature on joint hypermobility in women with lipedema where ≥67% of women have hypermobile joints. There is a suggestion that mast cell activation disease [53] or perhaps other inflammatory disorders contribute to hypermobile joints. Our data therefore support lipedema as a connective tissue disease whose gene or genes remain to be identified, or an inflammatory disease that not only that destabilizes joints.

## 5. Conclusions

This study introduces two intermediate stages of lipedema, 1.5 and 2.5. These additional stages should provide more accurate phenotyping of transitional patients, improved clinical documentation of progression, enhanced stratification for research and treatment planning, and allow for better alignment with imaging and biomarker development for objective disease tracking. Outcomes of this study support that the higher the lipedema stage, the higher the BMI, pain, swelling, and tissue heaviness, particularly in the thighs, calves, and lower back. It is also now clear that fat accumulation begins in the calves in most women with lipedema and spreads to the knees and feet, emphasizing the need for a comprehensive and even systemic assessment of all aspects of the body; altered cognition in women supports this systemic assessment. Total body water increases across stages, so that women with Stage 3 lipedema are at higher risk of developing lymphedema. And while muscle mass increases alongside fat, either as a compensatory mechanism to support excess tissue, or secondary to inflammation and swelling, mobility remains to be tested. Our study also points to inflammation and fibrosis as important areas of continued study. Phase angle may be a good non-invasive measure of inflammation in women with lipedema.

## Figures and Tables

**Table 1:** Demographics: stage, number, age and BMI of lipedema patients.

Lipedema Stages	N	Age	BMI
Stage 1	8	44.1 ± 3.9	33.1 ± 11.0
Stage 1.5	35	51.2 ± 10	28 ± 5.1
Stage 2	32	51.2 ± 11.1	34.3 ± 7.4
Stage 2.5	8	44.1 ± 3.9	39.2 ± 3.8
Stage 3	19	59.1 ± 11.2	47.4 ± 11.8

**Table 2:** Description of new half stages (1.5 and 2.5) and classic lipedema stages (1, 2 and 3). Summary of characteristic skin and hypodermal features across classic and newly proposed half-stages.

Stage	Tissue	
	Skin	Hypodermis
**1**	Smooth	Grainy to pearl-sized nodules
**1.5**	Indentations in the skin that affect the upper ½ or lower ½ of the leg anterior, lateral and posterior	Grainy to pearl-sized nodules
**2**	Indentations in the skin of the entire thigh, anterior, lateral and posterior	Grainy to pearl-to-walnut-sized nodules
**2.5**	Beginning of lobules in the tissue especially at the hip and around the knee; indentations in the skin of the entire thigh, anterior, lateral and posterior	Grainy, pearl-to-walnut-sized and larger nodules; lobules around the hips and knees; overhang over the elbow often present
**3**	Skin folds on the upper arms, abdomen, hips and knees; indentations in the skin of the entire thigh, anterior, lateral and posterior	Grainy, pearl-to-walnut-sized, and larger deformations; multiple lobules on upper arms, over elbows, on the abdomen, hips, thighs, knees and ankles

## Data Availability

Data are contained within the article and Supplementary Materials available from the authors upon request.

## Supplementary Materials

The following supporting information can be downloaded at: https://www.mdpi.com/article/10.3390/life15091397/s1, Figure S1: Correlation between TBW and EFC (a) and ICF (b).
