# Supplementary figures and images for: New Characterization of Lipedema Stages: Focus on Pain, Water, Fat and Skeletal Muscle
Source: Life (Basel). 2025 Sep 3;15(9):1397. doi: 10.3390/life15091397 (PMC12471503; doi:10.3390/life15091397)

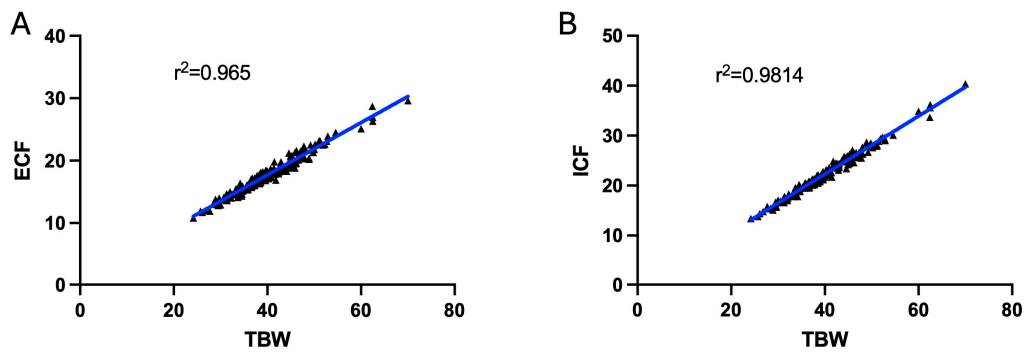

Figure S1: Correlation between TBW and EFC (a) and ICF (b).

Supplement: Supplementary file 1 [file life-15-01397-s001.zip › life-3778933-supplementary.pdf]
